# From Praising the Remedy to Eulogising the Patient: Cristóbal de Castillejo’s Satire of Guaiac in Early Modern Spain

**DOI:** 10.1093/shm/hkad072

**Published:** 2023-10-06

**Authors:** Ivana Bičak

**Keywords:** early modern medicine, satire, guaiac, Cristóbal de Castillejo

## Abstract

The article examines the contemporary satiric treatment of a new transatlantic drug, guaiac, in a sixteenth-century poem by the Castilian writer Cristóbal de Castillejo, entitled *En alabança del palo de las Indias, estando en la cura dél* (*In Praise of the Wood of the Indies, being under its treatment*). The article contributes to the body of scholarship on the history of medicine in general and the history of herbal medicine in particular. The investigation of the poem embraces historical contextualisation, early modern rhetoric and classical reception. The article demonstrates the two-way relationship between early modern satire and medicine, arguing that the special significance of satiric productions that engage with medical themes lies in the inventive combination of the literary reality and empirical reality.

In the last few decades, the history of medicine has taken into account social, economic and cultural aspects of historical medical developments. As the editors of a recent study on Spanish medical cultures put it, medicine constitutes ‘a significant matrix for the intersection of a wide range of cultural phenomena (political, literary, religious, or otherwise)’.^1^ Literature plays a significant role in narrating the history of medicine at a particular historical moment by painting a vivid picture of contemporary reactions to novel medical treatments and remedies. Literary writings offer an exceptional immediacy of first-hand experience and trace the enthusiasm and scepticism that frequently accompanied new medical discoveries and developments.

Within the literary category of medicine’s complex matrix, satire deserves to be propelled into view as a valuable source material for the history of early modern medicine.^2^ With its curious ‘extra-literary purchase on reality’,^3^ satire incorporates current affairs into its literary fabric, taking its inspiration from and reacting to the protagonists, actions and events in the empirical world. Its characteristic topicality provides illuminating details on contemporary medical phenomena, some of which may escape the more traditional source materials of the history of medicine. This is not to say that satire provides a faithful documentary reality, but rather that it offers a fresh vantage point for the exploration of past medical issues.

This article explores the contemporary satiric treatment of guaiac, a new transatlantic drug, in a sixteenth-century poem by the Castilian writer Cristóbal de Castillejo, entitled *En alabança del palo de las Indias, estando en la cura dél*^4^ (*In Praise of the Wood of the Indies, being under its treatment*).^5^ The article contributes to the body of scholarship on the history of medicine in general and the history of herbal medicine in particular.^6^ The following investigation of Castillejo’s satiric poem embraces historical contextualisation, early modern rhetoric and classical reception. The article demonstrates the two-way relationship between early modern satire and medicine, arguing that the special significance of satiric productions that engage with medical themes lies in the inventive combination of the literary reality and empirical reality.

‘Oh, doctor Herb, learned without Galen’,^7^ exults Francisco de Quevedo in his 1648 sonnet on the therapeutic possibilities of the tobacco plant. Quevedo’s verse reflects the medical pluralism of the early modern Spanish empire, where academic medicine rooted in Galenism coexisted, interacted and competed with extra-official healing practices.^8^ Moreover, the playful and satiric sonnet gives a glimpse into the influence of the New World *pharmacopoeia* on the medical and cultural landscape of the Iberian Peninsula, where novel remedies were gradually assimilated into the Galenic humoral system.

Guaiac wood (*Guaiacum officinale*) entered Spain by 1508 and affected both the medical and literary scene of the time. Its popularity spread like wildfire throughout early modern Europe, which was then in the grips of the French pox (also called *mal francese, morbus gallicus*, *bubas*, great pox, *morbus Neapolitanus*, *morbus curialis*, etc.). The wasting, highly contagious disease ‘struck everywhere, from palatial homes to insalubrious basement apartments, from the halls of powerful clerics to the sunbaked fields of farm laborers’, as Cristian Berco vividly describes it.^9^ In a 1498 poem that witnesses the earliest spreading of the French pox in Spain, Francisco López de Villalobos writes:

It was a pestilence ne’er to be found at all
In verse or in prose, in science or in story,
So evil and perverse and cruel past control,
Exceedingly contagious, and in filth so prodigal^10^ (III. 1–4)

The French pox caused atrocious symptoms that included festering pustules, joint pain, baldness, lost nose cartilage, broken bones and other painful symptoms. The cure was therefore desperately needed to relieve patients from all walks of life, from courtiers to prostitutes. The theory that Columbus and his crew had imported the disease when they returned from America in March 1493 (‘Indian measles’)^11^ made the appeal of guaiac so much the greater: according to the medieval doctrine, God had placed the remedies close to the diseases (*unde morbus*, *inde remedium*).^12^

Apart from receiving support from the theory of the American origin of the French pox, guaiac proved a gentler remedy than mercury, the staple treatment of the condition at the time. ‘A night with Venus, a lifetime with Mercury’, the popular adage went. Mercury could be applied externally as a salve, ointment, rub or plaster; it could also be taken internally. There were even ingenious anti-venereal underpants available in Italy, coated with a mercurial ointment on the inside.^13^ The problem with mercury, however, lay in its exceeding efficiency: the patient was not exactly whole after the treatment. Patients that were treated—or rather, poisoned—with mercury suffered terrible side effects, including uncontrollable salivation, tremors, corrosion of jaw bones, loss of teeth, cachexia (muscle loss), kidney damage and accompanying psychic disturbance. Many sufferers were therefore keen to discontinue ‘the Martyrdom of Mercury’, to use the title from the 1709 engraving in a treatise by John Sintelaer that depicts hospital patients treated for the French pox (Figure 1).

**Fig. 1:**
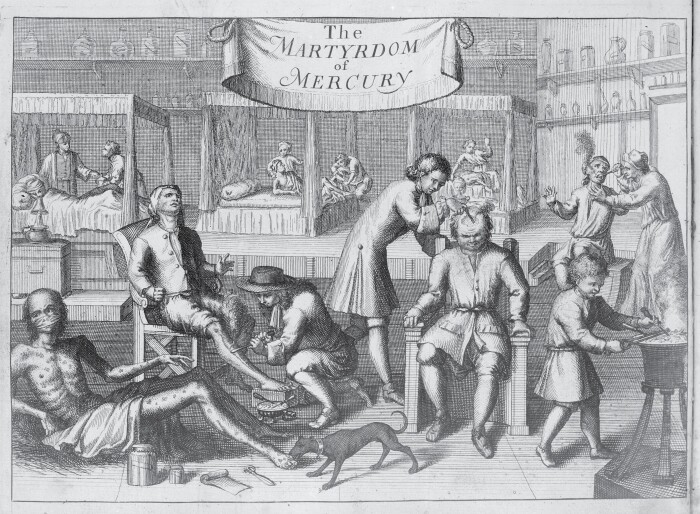
John Sintelaer, *The scourge of Venus and Mercury, represented in a treatise of the venereal disease* (1709). Wellcome Collection.

In *The scourge of Venus and Mercury*, the treatise where this print appears, Sintelaer wonders:

But what seems to be more amazing still, is, that after these unfortunate Creatures see themselves thus Infected, by the *Poisonous Fruits Venus* bestows upon those, that with an uncontrouled Desire will intrude themselves into her Labyrinths, they should have this additional Misfortune, to be entangled also in the most deceitful Snares of *Mercury*, whose Lash being so severe, as not only to tear the Flesh, but also to strike to the very Bones, their Case in stead of becoming better, is thus rendred much worse, to the accomplishment of their Destruction.^14^

Venus and Mercury, the cause and the treatment of the disease, respectively, are here presented as complementary in their deceptiveness and cruelty. Venus lures her victims with ‘*Poisonous Fruits*’ of carnal pleasures and leads them into her ‘Labyrinths’, while Mercury entangles them in his ‘most deceitful Snares’. Mercury pretends to be the remedy, but in reality it is an ‘additional Misfortune’, a flesh-tearing and bone-striking Lash.

Therefore, as a welcome alternative to the horrors of mercury, guaiac raised high hopes. In *Syphilis sive morbus gallicus* (1530), Girolamo Fracastoro hails the vital wood with these words:

*Iberian* Coasts you first were happy made
With this rich Plant, and wonder’d at its Aid;
Known now to *France* and neighbouring *Germany*
Cold *Scythian* Coasts and temp’rate *Italy*,
To *Europe*’s Bounds all bless the vital Tree.^15^

The religious overtones are no less conspicuous in Dr Gideon Harvey’s *Great Venus Unmasked* (1672), where the Portuguese, ‘receiving a miraculous cure from that sacred Wood, hung pieces of it in their Churches, and kneeling down before it, blessed God for his Mercy in discovering so sacred a Wood’.^16^ Guaiac was known under the names of *lignum sanctum* (holy wood) and *lignum vitae* (wood of life), phrases usually used to describe Christ’s cross. Ulrich von Hutten, the author of the most influential book on guaiac in sixteenth-century Europe, mentions that contemporary physicians call the wood ‘manus Christi’, ‘Apostolicon’, ‘gratia Dei’ or ‘Antidotum paulinum’.^17^ Meanwhile, Francisco Delicado’s frontispiece to his 1525 treatise on guaiac goes so far as to depict the Virgin Mary sprouting from the wood (Figure 2).^18^ Expectations therefore soared as ‘pocky’ patients consumed by mercury turned their hopeful gaze towards the new Wunderbaum,^19^ a heaven-sent and life-giving remedy. Between 1568 and 1608, more than 21 tons of guaiac reached Seville, the main port of entry for American drugs.^20^

**Fig. 2:**
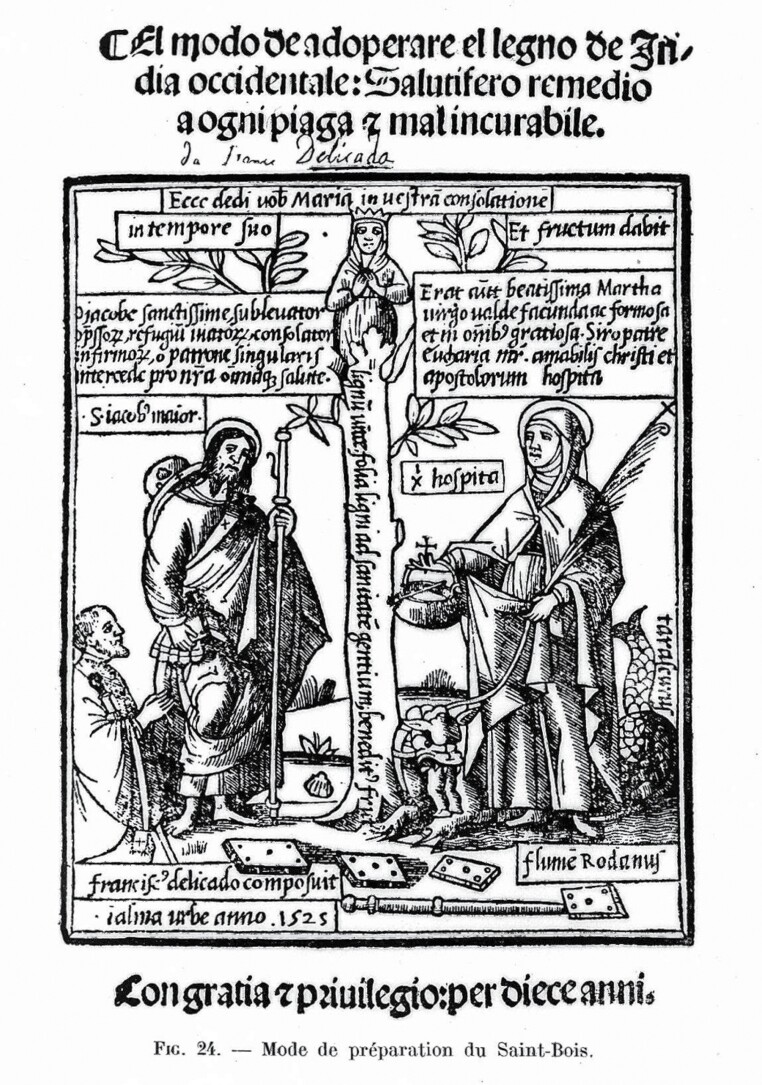
Frontispiece to Francisco Delicado, *El modo de adoperare el legno de India occidentale* (1525), as printed in Édouard Jeanselme, *Historie de la syphilis, son origine, son expansion: progrès réalisés dans l'étude de cette maladie depuis la fin du XVe siècle jusqu'à l'époque contemporaine* (1931). Wellcome Collection.

There are two major species of this wood, *Guaiacum officinale* and *Guaiacum sanctum.* The former is regarded as genuine guaiac. It can be found on nearly all islands of the West Indies, coastal regions of tropical North America, and parts of the northern coast of South America and its islands. The plant is a large evergreen with yellow flowers, and its wood is very dense and heavy. Its trunk is covered with a smooth grey bark. When imported, the wood came in the shape of logs stripped of bark. The early modern preparation process consisted of chipping the wood, weighing the chips and cooking them in a pot following the recipe, a process neatly illustrated in a late sixteenth-century copperplate engraving by Philips Galle after Jan van der Straet (Figure 3). Sometimes the wood was administered in the form of pills instead of decoctions.

**Fig. 3:**
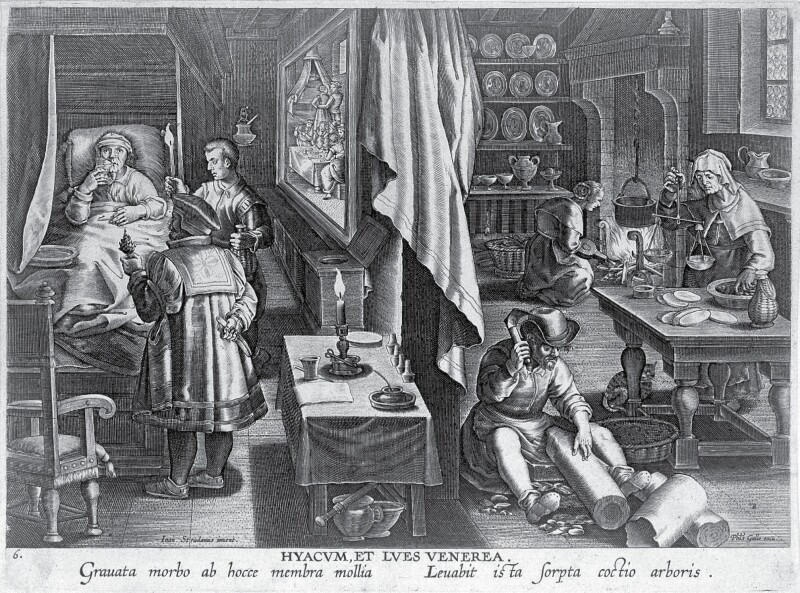
Line engraving by Philips Galle after Jan van der Straet, showing the preparation of the medicine guaiacum from a tree (right), and a man in bed suffering from syphilis, drinking a decoction of the medicine (left) (1600?). Wellcome Collection.

As guaiac entered Spain and made its way throughout Europe, it occasioned the establishment of ‘wood houses’, hospitals for the treatment of the French pox in Augsburg, owned by the banking family of the Fuggers. The Fuggers secured a monopoly on guaiac in exchange for a large loan that helped support the Spanish throne.^21^ Numerous medico-botanical treatises devoted to guaiac appeared in Latin and the vernacular.^22^ The enthusiasm for the new plant was boosted by observing the recovery of patients who switched their therapy from mercury to guaiac. In reality, the improvement was due to the fact that simply discontinuing mercurial therapy alleviated the symptoms.

Guaiac and other newly imported exotic drugs, including Santo Domingo balsam, sarsaparilla and ipecacuanha, met with a variety of responses, resulting from the tension between university-educated physicians and guild-based apothecaries. At the time, learned medicine in Europe relied on Galenism, a system in which the treatment of the patient and the prescribed diet and regimen depended on an individual’s age, constitution and temperament. Galenism presupposed a spectrum between health and illness, and the goal of the physician was to adjust the individual’s humoral balance. As Jon Arrizabalaga, John Henderson and Roger French have shown, for the medical establishment, the case of the French pox proved especially challenging.^23^ Here was a disease that looked more like an entity and that passed from one individual to another. It was therefore difficult to fit the pox into the theoretical apparatus of learned medicine. While the medical establishment was coming to grips with how to explain the new disease, empirics acted quickly. As the view of the French pox shifted towards the notion of ‘whole substance’, the empirics used guaiac as a *specific*, a cure aimed at a specific disease. In this ontological view of disease, bodies were seen as more or less interchangeable: a specific’s universal efficacy meant it would work on every person, regardless of their individual physiological make-up. Thus, the empirics’ promise of a swift and easy cure irritated the medical establishment, which relied on learning and method, as well as the physiological view of the body and the focus on an individual patient.

While guaiac was being quickly adopted by empirics,^24^ its use belonged to a broader debate between traditional Galenists and medical humanists regarding the exotic *materia medica*. Medical humanism, which arose at the end of the fifteenth century, was critical of errors in Latin medical manuscripts that had been translated from the works of Arab physicians. Medical humanists produced new translations of Greek medical treatises, challenging orthodoxy and advocating new therapeutic practices. Such an approach encouraged experimentation and focused on simples (remedies derived from just one plant, animal or mineral) rather than Galenic compounds. As a result, medical humanists readily accepted the American *materia medica*, including guaiac.^25^

Hutten himself complained of the physicians’ hostile stance towards guaiac, saying that ‘the phisitions wold not alowe it, perceyuynge that theyr profite wolde decaye thereby’.^26^ At least in the beginning of the importation of guaiac, profit certainly played a major role. As Harold Cook has argued, the shift from the individual to the unique body suited the growing commercialisation of the medical marketplace.^27^ Anyone afflicted by a condition could be a potential purchaser and consumer of the specific cure. The unprecedented number of potential consumers meant that trade in medical specifics was attractive to Casa de la Contratación, an institution established by the Crown in Seville to regulate the commerce between Spain and the Americas. As a consequence of this economic interest, physicians and hospitals received official requests for remedy trials. However, these trials were not without their opponents. In the case of the popular Santo Domingo balsam, a Hispaniola physician, Licenciado Barreda, contended that the balsam propagated by the crown was fake and that it posed a health risk.^28^ Yet the crown’s answer to that complaint was to continue the trials and observe the outcomes.

Interestingly, the new cures were gradually implemented into the Galenic system. The Venetian physician Nicolo Massa, for example, explained the French pox in terms of an excess of cold phlegm that originated in the liver.^29^ Physicians argued that the doses should be adjusted according to the patient’s individual constitution and that regimen and diet should also play a role in the treatment. In the case of guaiac, the sweat therapy and dietetic regime certainly played a significant role, but these were not determined individually.

In the complex medical context of the sixteenth century, the dynamic medical marketplace and the officially encouraged experimentation raised new hopes for desperate patients, but occasionally also dashed them. In the literary realm, guaiac and other American medicinal plants, especially sarsaparilla, spurred a satiric production that tried to come to its own terms with the novel remedies and their influence on European medical practice.^30^ Satire was a natural response to these developments as its generic identity depends on mediating the empirical world. Moreover, satire is firmly intertwined with the art of medicine and healing since it frequently uses medical rhetoric and imagery to diagnose the society’s ills.^31^ Therefore, its interest in contemporary medicine is an expected literary development.

In the first half of the sixteenth century, guaiac occasioned a long, 108-line poem with a correspondingly long title, *En alabança del palo de las Indias, estando en la cura dél* (*In Praise of the Wood of the Indies, being under its treatment*). Written by Cristóbal de Castillejo (1490?–1550), this mock encomium on guaiac is one of the earliest satiric compositions dedicated to an American product. It deserves to be translated into English as it not only offers an intriguing insight into a specific episode in the history of European medicine, but also shows how novel medical developments affected contemporary literature itself.

*In Praise of the Wood of the Indies* could be said to belong to what José Ignacio Díez Fernández has called ‘the poetics of syphilis’,^32^ but it would be better to call it the poetics of the French pox. Although there are many historical studies that identify this disease as syphilis, retrospective diagnosis is an anachronistic historiographical practice. In the analysis of an infectious disease, it is impossible to reconstruct historically the interactions between the host, parasite and the environment. Such a reconstruction is also problematic as the early modern medical paradigm is incommensurable with our own.^33^ In any case, there is an entire body of poetry dedicated to this disease in early modern Spanish literature—and there is also abundant accompanying scholarship.^34^ After all, the French pox was ‘one of the merry diseases in the carnivalesque system’,^35^ and proved a fertile ground for the literary grotesque. However, instead of focusing on the aspect of the ‘venereal epidemic’^36^ in poems such as Castillejo’s, this article directs the spotlight at the problem of new and foreign medicine.

Castillejo’s poem is a suitable arena for exploring the ‘new’ and ‘foreign’, owing to the author’s firm stance on other cultures and languages. Castillejo left his small city of Ciudad Rodrigo (Salamanca) at the age of fourteen and spent much of his life at court in Spain and Vienna as a secretary and diplomat. Despite his cosmopolitanism, the proud Castilian was an avid adversary of cultural contamination, in particular the importation of Italian metrical innovations into Spanish poetry.^37^ How does his proclaimed rejection of ‘new’ and ‘foreign’ literary developments square with the reception of Amerindian guaiac? What implications does this ‘new’ and ‘foreign’ medicine have for literature in general, and satire in particular? The answers to these questions lie not only in the poem’s content, but also in its generic attributes.

*In Praise of the Wood of the Indies* belongs to the rich literary tradition of mock encomia.^38^ This is a subgenre of the classical encomia or *laudationes*, dedicated to gods, heroes, countryside, abstract virtues and similar dignified themes.^39^ However, unlike these laudatory speeches, mock encomia take up *infames materias*, *sive quis mavult dicere inopinabiles*, *quas Graeci ἀδόξους ὑποθέσεις appellant* (‘ignoble subjects, or if you prefer, unexpected ones, ἄδοξοι ὑποθέσεις, as the Greeks call them’).^40^ This is where the genre derives its alternative name, adoxography (*ἄδοξος* = inglorious, disreputable). Arthur Stanley Pease has famously called the subjects of adoxographic praise ‘Things without Honor’, that is, ‘persons or objects in themselves obviously unworthy of praise, as being trivial, ugly, useless, ridiculous, dangerous, or vicious’.^41^ If Castillejo’s poem is a mock encomium, then guaiac is, by implication, useless and unworthy of praise. However, as this article demonstrates, the situation is far more complicated than that and nicely mirrors the complex reception of guaiac in both medical and extra-medical communities of the period.

Mock encomium is frequently also called paradoxical encomium (*παράδοξος* = contrary to expectation).^42^ The genre’s literary pedigree is so rich that critics have resorted to terms such as ‘an epidemic of apparent nonsense’^43^ and ‘*paradoxia epidemica*’^44^ in the early modern period. Since ancient times, the subjects have included everything from parrots to quartan fever and laziness.^45^ The choice of an unexpected subject is supposed to show off the orator’s skill and to dazzle the audience. Crucially for our poem on guaiac, paradoxical encomia have ‘duplicity built into it’.^46^ For this cheeky genre raises the question: ‘can a thing unpraisable in fact be praised? If it can, then it is not unpraisable; if it cannot, then a vast number of pieces of paradoxical prose do not exist’.^47^ Castillejo’s poem relies on the concept of duplicity, lying and telling the truth at the same time.

Castillejo’s mock encomium has been described as ‘an ode to guaiac’^48^ and as ‘true praise converted into the burlesque’.^49^ Elsewhere, the poem’s tone has been deemed ‘scurrilous; Castillejo wastes a great display of learning and a few passages of great beauty on an unworthy theme’.^50^ There seems to be a good deal of bewilderment as to the nature and meaning of the poem. The remedy for this confusion lies in close-reading the poem against the backdrop of contemporary medical developments. The unstable position of guaiac in the poem brilliantly reflects the contemporary mixture of exhilaration and scepticism when it came to the novel American import. The most famous opponent of guaiac in Europe was Paracelsus, who refused to believe in its miraculous properties and proclaimed it to be useless.^51^ The debate as to the efficacy of guaiac lasted for decades until the remedy’s popularity finally faded.

The poem is written in *novenas*, stanzas of nine verses with the rhyme scheme *abbaccddc*. The number of syllables in a line is not strictly defined in a *novena*—accordingly, the feet vary in Castillejo’s poem. In terms of content, the fifth line belongs to the first part of the stanza; in terms of rhyme, however, it belongs to the second part: [abba[c]cddc]. In this sprightly way, the stanza’s two constituent parts are compactly joined together, with the second part usually explaining or illustrating the first part.

Castillejo opens his poem with an invocation, followed by a foray into linguistic and national issues:

*Guayaco si tú me sanas,*
* y sacas d’estas pendencias,*
* contaré tus eccelencias*
* y virtudes soberanas*
* dulcemente:*
* no por estylo eloquente,*
* ni en lengua Griega, o Romana,*
* sino por la Castellana,*
* qu’es bastante y sufficiente.*
*Que caso que la Latina*
* tenga más autoridad,*
* no ay aquí necessidad*
* de eloquencia peregrina,*
* y que la aya*
* No es honra nuestra que caya*
* tu loor en tanta mengua,*
* que le calle nuestra lengua,*
* y la agena te le traya.* (1–18)

Guaiac, if you cure me
and release me from these troubles,
I will recount your merits
and sublime virtues
sweetly;
not in an eloquent style
nor in Greek or Latin,
but in Castilian,
which is much and enough;
admitting that Latin
has more authority,
there is no need here
for extraordinary eloquence;
and although it has authority,
it is not an honour to me
that your praise be reduced,
that our language does not mention it,
and that a foreign language carries it to you.

Already the first verse threatens to sabotage the poem’s title and its self-proclaimed genre: this will be an *alabança*, or *loor* (praise) only in the case that guaiac proves to be an efficient cure. Only then will the poet tell of its excellencies and sublime virtues. The first stanza therefore presents a *quid pro quo* situation, a bargain on the table. As for linguistic nationalism, it is interesting to compare Castillejo’s decisive rejection of foreign languages with the fact that the poem does not escape classical authority as Roman literature and culture infiltrate its textual tissue. Moreover, Castillejo’s claims to Spanish originality are undermined by the existence of the Italian tradition of humorous poetry where ‘a pathetic mixture of the jocose unites with the biographical background of the sufferer’.^52^ Indeed, Spain imported the entire tradition of the paradoxical encomium from Italy.

In the following stanza, Castillejo quickly concludes that guaiac will surely prove to be worthy of praise. He refers to Cato the Elder’s tribute to cabbage in *De agricultura*:

*Si halló Marco Catón*
* causa de alabar la berça,*
* más la terne yo por fuerça,*
* de celebrar con razón*
* la virtud*
* de un árbol que da salud,*
* do se tiene por perdida,*
* y a las vezes buelve en vida*
*el mal de la juventud.* (19–27)

If Cato the Elder found
reason to praise the cabbage,
the more had I, inevitably,
to celebrate with good reason
the power
of a tree that gives health
where it is considered lost,
and sometimes returns to life
the illness of youth.

Cato asserted that *Brassica est quae omnibus holeribus antistat* (‘It is the cabbage which surpasses all other vegetables’)^53^ and argued for its medicinal virtues.^54^ However, at the mention of this vegetable, an educated sixteenth-century reader would simultaneously think of the alternative Roman tradition, in which cabbage was unmistakably seen as poor people’s food. The stinking *pallidus caulis* (‘faded cabbage’) is contrasted with the delicacies served to the rich in Juvenal’s fifth satire.^55^ Moreover, in the Spanish baroque tradition, vegetables such as cabbage had ‘a marked comic charge since they are cheap food that provoke gases’.^56^ With a tongue in cheek, Castillejo thus compares the miraculous cure of the New World with a lowly vegetable disguised as a celebrated remedy. The conjunction ‘if’ at the beginning of the passage is telling since it suggests that cabbage is a ‘Thing without Honour’.

The praise soars to hyperbolic heights when it is claimed that the discovery of guaiac would be reason enough for the conquest of America:

*Aunque no diera más parte*
* de gloria a nuestra nación,*
* la conquista de Colón,*
* que ser causa de hallarte.*
* es tamaña,*
* tan divina, tan estraña,*
* ésta que por ella sola,*
* puede muy bien la Española*
* competir con toda España.* (28–36)

Were Columbus’ conquest alone
to have given our nation
no greater glory
than having discovered you,
that is huge,
so divine, so singular,
because, for that cause alone
Hispaniola can well compete
with the entire Spain.

Hyperbole is a figure of speech that uses exaggeration for rhetorical effect. Every hyperbole rests upon the process of expansion. In this hyperbole of worth,^57^ we see an island in the Caribbean archipelago loom greater than the entire Spanish Empire. A world power that extends far beyond the Iberian Peninsula shrinks in worth in comparison to Hispaniola. This augmentation of Hispaniola is paradoxical since the discovery of guaiac depends precisely on Spain’s conquest of Hispaniola. The *amplificatio* of grand adjectives (*tamaña*, *divina*, *estraña*) gives hyperbole wings in these verses. Castillejo uses ‘the Baroque’s most Baroque figure’,^58^ as Christopher D. Johnson calls this figure of excess, in order to break with the possible and create ‘a deviant reality’.^59^

To show this new, off-the-beaten-track reality (*devius* = literally ‘out of the way’), Castillejo extends his poetic gaze to the opposite side of the globe as ships laden with oriental spices make their way towards Europe:

*Abaxen los Orientales*
* la presunción, y la vela,*
* con sus clavos y canela,*
* y otros mil árboles tales*
* que ay entrellos,*
* odoríferos y bellos,*
* en aquel vergel de Apolo,*
* que nuestro Guayaco solo*
*vale más que todos ellos.* (37–45)

May the Orient take down
its conceit and sails,
with its cloves and cinnamon,
and another thousand of such trees
that they have,
fragrant and lovely,
in that orchard of Apollo;
since our guaiac alone
is worth more than all of these.

In this evocative vignette, Apollo’s vast orchard of fragrant and beautiful trees fades in comparison with guaiac. In a witty hyperbolic play of proportions and numbers, a single bark trumps thousands of plants. Castillejo’s deviant reality goes against the empirical reality, in which guaiac did not miraculously obliterate the market that was inundated with exotic plants, herbs, and spices.

The poem claims that the reason for guaiac’s supremacy can be attributed to the fact that other plants are effective only as parts of medicinal compounds:

*Todas las plantas preciosas*
* de saludables secretos,*
* comunican sus effetos,*
* ayudadas de otras cosas.*
* De manera,*
* que la que más se esmera,*
* muy poquitas vezes sana*
* la dolencia más liviana,*
*si no le dan compañera.* (46–54)

All the precious plants
of salutary secrets
impart their effects
with the help of other things;
such that
even the plant that tries the hardest
very rarely cures
the lightest of ailments
without a companion.

Also known as polypharmacy, the Galenic compounding of medicines included mixing different simples into a single, compound medicine. The process required expertise in order to maximise the benefits of the participating simples. In Paula S. DeVos’ words, ‘authors in the Spanish pharmaceutical tradition viewed compounding as the culmination of the apothecary’s work’.^60^ Hutten himself ridiculed the system of polypharmacy and the principle of the ‘shotgun-therapy’.^61^ In this system, all the other plants are too weak to work their effects without a companion so they need to participate in ‘poticary compositions’.^62^ Not so guaiac:

*Mas vos Guayaco gentil*
* descubierto nuevamente,*
* por bien común de la gente,*
* y remedio de cien mil:*
* sin escudo,*
* y a solas contra el más crudo*
* mal que en el mundo se halla,*
* do la medicina calla,*

*entrays en campo desnudo.* (55–63)

But you, noble guaiac,
recently discovered
for the common good of the people
and remedy of a hundred thousand,
without a shield
and alone against the harshest
disease that there is in the world,
where medicine is silent,
you enter the field unarmed.

Castillejo again employs a hyperbole of number (*cien mil*) to emphasise guaiac’s immense potential. In a wonderfully mock-epic image, we see a piece of wood become a brave warrior that enters the desolate medical battlefield without a shield, that is, without the help of another simple. Where *all* medicine fails, guaiac alone seems to emerge victorious. ‘*Sin escudo*’ also has a double meaning since *escudo* also designated a gold coin. The pun points at the supposedly high price of guaiac which leaves the patient penniless. ‘*Entrays en campo desnudo*’ is also a witty allusion to the metaphor of love as a battle and a reference to the way in which the disease is contracted.

The following lines continue the *enumeratio* of plants whose qualities are outstripped by guaiac. This redundancy does not have a mere ornamental function; rather, it serves to inculcate the idea of guaiac’s supremacy. Johnson has dexterously applied the procedure used in the backup of an engineering or computer system to the Renaissance poetics of enumeration: ‘any single component or system (n) will sooner or later fail. Given this, typically engineers add another similar or identical component (n+1) to carry the load should it become necessary. N+2 means that there is a backup for the backup’.^63^ Castillejo thus makes a backup for the backup, multiplying his herbal examples, until he reaches n+1006. To the cinnamon, clove, and a thousand other plants Castillejo adds the great four:

*Tiene el cedro por su altura*
* la palma por su grandeza,*
* el laurel por su nobleza,*
* y el ciprés por su hermosura*
* eccelencia,*
* Mas llegada en competencia*
* la de todos con la tuya,*
* de tu virtud a la suya,*
*ay muy grande differencia.* (64–72)

The cedar has its height,
the palm has excellence
because of its size,
laurel because of its nobility
and cypress because of its beauty:
but, come the competition
of all the others with you,
between your efficacy and theirs
there is a very large difference.

The four trees stem from the rich tradition of classical and Biblical plant imagery. Each verse links the tree with its dominant quality. Since the four trees stand for majestic height (cedars were frequently connected with royalty), greatness, nobility and beauty, it is difficult to imagine other plants surpassing them. Yet, even when taken together, they can be no match for guaiac.

From the royal height of cedars, the verses unexpectedly and bathetically sink to the ordinariness of a walnut tree:

*No me burlo yo contigo*
*como el otro del nogal* (73–74)

I am not joking with you
like another one does with the walnut tree,

‘The other’ refers to Ovid as the author of *Nux*, an elegiac poem in which a roadside walnut tree complains about its merciless treatment at the hands of the passers-by. In this exercise in forensic rhetoric (legal discussion of past actions), the talking tree presents a legal case against being pelted with stones and robbed of its fruit. Ovid’s poem is a frequent reference point for Renaissance mock encomiasts as it provides a good example of playful parody.^64^

Castillejo’s reference to the *Nux* and his explicit denial of any paradoxical purpose in his own poem immediately raises suspicions. A paradox inhabits the space ‘*entre burlas y veras*’ (‘between joke and truth’).^65^ Castillejo’s mere mention of the verb that is etymologically connected to the burlesque (*burlar* = to mock, flount, joke) casts a shadow of doubt over the poem’s supposed sincerity.

The magical plant is treated as a deity as Castillejo begs for health:

*Pero ruégote y suplico,*
* que alargues en mi tu mano,*
* porque pueda verme sano,*
*pues no me puedo ver rico*. (91–92)

But I beseech and beg you
to extend your hand to me,
so I could see myself healthy
since I cannot see myself rich.

As with ‘sin escudo’ in line 59, Castillejo is here alluding to the presumably high cost of guaiac in the sixteenth century. Since many New World medicines were probably exchanged informally and sometimes immediately exported further from Seville,^66^ it is difficult to determine the exact retail prices at the time. Moreover, the Spanish Price Revolution brought about an inflation of consumer prices in the sixteenth century, so the price would have varied from decade to decade. The cost of guaiac also varied according to the quality of the wood and the demand at a given time. However, at the beginning of the sixteenth century, guaiac may well have sold for a high price owing to its novelty.^67^ Even in late sixteenth-century Florence one course of guaiac therapy could cost between 2 to 13 per cent of annual earnings of an unskilled construction worker.^68^ And even with the price drop that followed the fall in guaiac’s popularity in Europe at the end of the century, the treatment with guaiac could still be a costly affair since it required firewood for the accompanying heat therapy (more on that below). A good case study is a 1527 set of detailed financial records kept by a victim of the French pox, one Johannes Sinapius.^69^

After Castillejo accepts the idea that guaiac will drain him of his financial resources, he addresses the plant with the final exasperated invocation:

*O Guayaco*
*enemigo del Dios Baco,*
*y de Venus y Cupido,*
*tu esperança me ha traydo*
*a estar contento de flaco.* (95–99)

O, guaiac!
Enemy of the god Bacchus
and of Venus and Cupid,
from being feeble, hope in you
has made me content.

The deification of guaiac occurs through its comparison with the Roman gods of wine and love, seen here as the originators of his condition. Interestingly, nowhere in his poem does Castillejo mention the name of the condition, implying that sexual contact was recognised as a significant mode of contagion by this time, as is also implied in *Entrays en campo desnudo* in line 63. Below, the speaker will refer to being repentant of his loves, enforcing this interpretation. Indeed, the French pox had been so inextricably linked to ‘the act of Venus’ that it received the name *lues venerea* (‘venereal disease)’.^70^ And since *Sine Baccho friget Venus* (‘Without Bacchus, Venus freezes’),^71^ alcohol was seen as an enabler of risky amorous encounters. Abstinence from sex was therefore paramount in the treatment with guaiac. In his renowned survey of the *materia medica* of the New World, *Historia medicinal de las cosas que se traen de nuestras Indias Occidentales* (1565–1574), Nicolás Monardes warned that the sick man should not ‘returne to tumble in the same bosome, where he tooke the firste [attack]’.^72^ Wine was allowed, but only in small quantities and then diluted with water.

The reference to the speaker’s feeble condition has two playful meanings. Apart from signifying the predicted loss of money, it points to the unpleasant physical reality of the guaiac treatment. Here, at the end of the poem, comes Castillejo’s unpleasant twist. All the praise previously given to guaiac ends in this vivid vignette:

*Mira qu’estoy encerrado*
* en una estufa metido,*
* de amores arrepentido,*
* de los tuyos confiado.*
* Pan y passas,*
* seys, o siete onças escassas,*
* es la tassa la más larga,*
* agua caliente y amarga,*
*y una cama en que me assas.* (100–108)

Look how I am locked up
placed inside a stove,
repentant of the loves
and trusting in your loves.
Bread and raisins
barely six or seven ounces
is the biggest portion I take,
hot and bitter water
and a bed in which I roast.

The patient is anything but free (he is ‘*encerrado*’). He is boiling in a hot bed in a hot chamber and subsisting on a meagre diet. Once again, Castillejo uses numbers and proportions for satiric effect: the lyrical subject of the poem is given scarcely ‘*seis ó siete onzas*’ (‘six or seven ounces’) of bread and raisins.

The patient’s ordeal in the poem is in accordance with Hutten’s detailed instructions as to how the guaiac treatment should proceed. Firstly, ‘The pacient muste be kepte in a close chambre, withoute ayre or wynde, where fyre muste be nourished contynually’.^73^ Secondly, ‘his meate must be dimynished’^74^ since ‘truly this medicine requirethe, that the pacient be made as thynne with hunger as maye be possible’,^75^ just like the patient in Castillejo’s poem. Hutten says that some prescribe the strict regimen of little chicken broth with bread for lunch, and a few raisins and an ounce of bread for dinner.^76^

Once the patient is starved in this way and purged, he is to take the first, stronger decoction of guaiac at 5 am and 7 pm daily for 4 days.^77^ After consummation, he is to undergo what Hutten admits is ‘the hardest thynge in all this cure’^78^: heat therapy. The patients were frequently covered hot with clothes for 3 to 4 hours. Hutten, however, warns against forced sweating, speculating that ‘through the operation of Guaiacum, he shal sweate inough’.^79^ After 15 days, the patient is purged and little more food is allowed, to be taken with the second decoction. Near the end of the treatment the patient is purged again and takes the decoction for 4 or 6 days more. The course of the entire treatment should last for at least 30 days. In all this time, the patient should remain in the same heated, air-tight room.

Therefore, although guaiac itself was a milder remedy than mercury, it included harsh ancillary treatments. Guaiac even acquired the name of ‘Diet woode’ in the first English Herbal of 1568.^80^ Accordingly, the poem that began on a flying, euphoric note with a promise of future health descends bathetically to a stifling scene of misery and suffering. ‘The Martyrdom of Mercury’ has only been substituted with ‘the Martyrdom of Guaiac’, with dubious results.

As we have seen, the poem relies on the Renaissance principle of *serio ludere* (‘playing seriously’) to explore the potential of a novel American medicinal plant. From a soaring, encomiastic mode enabled by hyperbole the poem regularly sinks into bathos (rhetorical anticlimax consisting in a fall from the sublime to the ridiculous): holy wood is compared with cabbage and walnut, and the patient is slowly reduced to a wretched state. The deviant reality where every other spice, herb or tree loses its value when compared to guaiac does not come into being. The poem suggests the supremacy and divinity of guaiac but fails to provide concrete evidence.

As a mock encomium that is fuelled by the concept of duplicity, *In Praise of the Wood of the Indies* relies on the simultaneous acceptance and rejection of the new and foreign medicine and of foreign literary traditions. The poem is itself a product shaped by the contemporary cultural reception of guaiac: the mock-encomiastic mode is dictated by the plant’s unrealised potential. Guaiac is given the dual role of a hero and satiric object—it is paradoxically a ‘Thing with and without Honour’, worthy and unworthy at the same time. Just as it accepts and rejects guaiac, the poem rejects Italian influence while decidedly belonging to an Italian genre. By presenting guaiac as an epic warrior and bringing it into relation with Roman deities, the poem participates in a ‘burlesque degradation of mythological themes in the Baroque period’.^81^

As we have seen, typical subjects of early modern paradoxical encomia are ordinary or lowly. Therefore, by the mere virtue of being the subject of Castillejo’s poem, exotic guaiac becomes endowed with unremarkable commonness. Due to generic conventions, the reader immediately knows that guaiac’s lofty flight will end in an embarrassing fall. In the empirical world of the sixteenth century, the high-flown expectations indeed exceeded guaiac’s actual performance, finally proving it to be unworthy. The holy wood’s popularity faded by the latter half of the sixteenth century. The poem that begins as a panegyric of guaiac ends as a satiric eulogy of the patient, in which Castillejo points at the uncertainty of guaiac’s potential to leave the bounds of its *honorificabilitudinitas*,^82^ ‘the state of being able to achieve honours’.

As in Quevedo’s sonnet about Dr Herb quoted at the beginning, in Castillejo’s poem guaiac takes the place of the physician. This substitution suggests the sheer power and prevalence of guaiac in the treatment of the French pox in early sixteenth-century Spain. The potency of satiric texts such as Castillejo’s lies in their questioning of the benefits of the new pharmacopoeia. In his satire, we see a vivid reflection of the contemporary reception of transatlantic drugs, influenced by the tensions between learned physicians on the one hand and medical humanists and empirical practitioners on the other. The promised deviant reality in which guaiac would prove to be a miracle cure was always just within reach, but never fully realised. More generally, Castillejo’s rich poem shows that the entangling of medicine with literature makes satire an animated and illuminating source for the history of medicine.

## Footnotes

1 John Slater, Maríaluz López-Terrada and José Pardo-Tomás, eds, *Medical Cultures of the Early Modern Spanish Empire* (Farnham: Ashgate, 2014), 2.

2 For an excellent overview of the diverse methodologies and source materials available to historians of early modern medicine, see Thomas Rütten, ‘Early Modern Medicine’, in Mark Jackson, ed., *The Oxford Handbook of the History of Medicine* (Oxford: Oxford UP, 2011), 60–81.

3 Frederick Jones, *Juvenal and the Satiric Genre* (London: Bristol Classical Press, 2007), 117.

4 Accentuation in the text of the poem has been modernised to avoid misunderstanding. Also, ‘v’ has been substituted for ‘u’ according to the phonetic value.

5 The date of creation is unfortunately unknown. The poem was published posthumously in *Las Obras de Christoval de Castillejo* (Madrid: Pierres Cosin, 1573), 367–71. I will be quoting the poem from this edition.

6 Some representative contributions to the field of the history of herbal medicine include Juan R. Sanchez-Ramos, ‘The Rise and Fall of Tobacco as a Botanical Medicine’, *Journal of Herbal Medicine*, 2020, 22, https://doi.org/10.1016/j.hermed.2020.100374; Elaine Leong, ‘“Herbals she peruseth”: Reading Medicine in Early Modern England’, *Renaissance Studies*, 2014, 28, 556–78; Patrick Wallis, ‘Exotic Drugs and English Medicine: England’s Drug Trade, c. 1550–1800’, *Social History of Medicine*, 2012, 25, 20–46.

7 ‘*O Doctor ierba, docto sin Galeno [...]*’, in Francisco de Quevedo, ‘Al Tabaco en polvo, Doctor a pie’, in *El Parnasso Español* (Madrid: Alberto Diego Diaz de la Carrera, 1648), XIII, 1, 421.

8 See María Luz López-Terrada, ‘Medical Pluralism in the Iberian Kingdoms: The Control of Extra-academic Practitioners in Valencia’, *Medical History – Supplement*, 2009, 29, 7–25.

9 Cristian Berco, *From Body to Community: Venereal Disease and Society in Baroque Spain* (Toronto: University of Toronto Press, 2016), 23.

10 Francisco López de Villalobos, *The Medical Works of Francisco López de Villalobos*, George Gaskoin (trans) (London: John Churchill and Sons, 1870).

11 ‘*Sarampion* [sic] *de las Indias*’, in Nicolas Monardes, *Historia Medicinal* (Seville: Alonso Escrivano, 1574), 13.

12 ‘*es tanta la clemencia divina, que adonde quiera que permite por nuestras culpas nuestros trabajos, allí a par dellos quiere que estén los remedios con su misericordia*’, in Gonzalo Fernández de Oviedo, *Historia general y natural de las Indias, vol. 1* (Seville: 1535), II, 14. ‘Divine clemency is so great that wherever it permits us to suffer afflictions for our sins, it mercifully places remedies for them’. Trans. mine.

13 John Parascandola, ‘From Mercury to Miracle Drugs: Syphilis Therapy over the Centuries’, *Pharmacy in History*, 2009, 51, 14.

14 Sintelaer, *The scourge of Venus and Mercury* (London: G. Harris, 1709).

15 Girolamo Fracastoro, *Syphilis sive morbus gallicus* (Verona: Stefano Nicolini da Sabbio, 1530), trans. Nahum Tate under the title *Syphilis: or, A Poetical History of the French Disease* (London: Jacob Tonson, 1686), 83.

16 Gideon Harvey, *Great Venus Unmasked* (London: Nathaniel Brook, 1672), 135.

17 Ulrich von Hutten, *De guaiaci medicina et morbo gallico liber unus* (Mainz: Jan Scheffer, 1519), Ch. VI.

18 Francisco Delicado, *El modo de adoperare el legno de India occidentale* (Rome: 1525; Venice: 1529).

19 Guaiac is named Wunderbawm in Johann Wittich’s treatise *Von dem Ligno Gvayaco, Wunderbawm* (Leipzig: 1592).

20 Pierre Chaunu and Huguette Chaunu, *Séville et l’Atlantique (1504–1650): Première Partie: Partie Statistique: Le Mouvement des navires et des marchandises entre l’Espagne et l’Amérique de 1504 à 1650* (Paris: S.E.V.P.E.N., 1956), 1027–29.

21 See John L. Flood and David J. Shaw, ‘The Price of the Pox in 1527: Johannes Sinapius and the Guaiac Cure’, *Bibliothèque d’Humanisme et Renaissance*, 1992, 54, 691–707.

22 For example, Delicado, *El modo de adoperare el legno de India occidentale*; Alphonsus Ferrus, *De ligni sancti multiplici medicina, et vini exhibitione* (Basel: 1538); Lorenz Fries, *Ein clarer bericht yetzt nüw von dem holtz Guaiaco* (Strasbourg: Johann Grüninger, 1529); Johann Wittich, *Von dem Ligno Gvayaco, Wunderbawm* (Leipzig: 1592); Max H. Frisch, ed., *Nicolas Pol, Doctor, 1494, with a Critical Text of His Guaiac Tract* (New York: Herbert Reichner, 1947).

23 Jon Arrizabalaga, John Henderson and Roger French, *The Great Pox: The French Disease in Renaissance Europe* (New Haven: Yale UP, 1997).

24 See Teresa Huguet-Termes, ‘New World Materia Medica in Spanish Renaissance Medicine: From Scholarly Reception to Practical Impact’, *Medical History*, 2001, 45, 359–76.

25 For an excellent discussion of medical humanism and medical botany in the sixteenth century, see Sean David Parrish, ‘Marketing Nature: Apothecaries, Medicinal Retailing, and Scientific Culture in Early Modern Venice, 1565–1730’ (PhD thesis, Duke University, 2015).

26 Ulrich von Hutten, *Of the Wood Called Guaiacum*, Thomas Paynel (trans) (London: Thomas Bertheletus, 1536), 11.

27 Harold J. Cook, ‘Markets and Cultures: Medical Specifics and the Reconfiguration of the Body in Early Modern Europe’, *Transactions of the Royal Historical Society*, 2011, 21, 123–45.

28 See Antonio Barrera, ‘Local Herbs, Global Medicines: Commerce, Knowledge, and Commodities in Spanish America’, in Pamela Smith and Paula Findlen, eds, *Merchants and Marvels: Commerce, Science, and Art in Early Modern Europe* (New York: Routledge, 2002), 163–81.

29 Nicolo Massa, *Liber de morbo gallico* (Venice: Bindoni and Pasini, 1527).

30 Agnolo Firenzuola, ‘In lode del legno santo’, in *Le Rime* (Florence: 1549), 118–20, and *Intorno alla sua malattia* (1533). Fernando Mejía de Guzmán, *La Zarzaparrilla* (ca. 1590) and Anastasio Pantaleón de Ribera, ‘Ad excelentísimo señor Duque de Lerma’. An extensive fragment of Guzmán’s *La Zarzaparrilla* has been preserved, with the introduction lacking. Guaiac was well known to London audiences, as can be seen from Ben Jonson’s *Volpone* (London: 1606), George Chapman’s *The Widdovves Tears* (London: John Browne, 1612), and John Lacy’s *The Dumb Lady* (London: Thomas Dring, 1672).

31 On the tight link between satire and medicine, see Sari Kivistö, *Medical Analogy in Latin Satire* (New York: Palgrave Macmillan, 2009).

32 José Ignacio Díez Fernández, *La poesía erótica de los Siglos de Oro* (Madrid: Ediciones del Laberinto, 2003), 286–87.

33 See Jon Arrizabalaga, ‘Infectious Diseases in Historical Perspective: French Pox *Versus* Venereal Syphilis’, *On_ Culture: The Open Journal for the Study of Culture*, 2021, 11, 2–15; Claudia Stein, *Negotiating the French Pox in Early Modern Germany* (Aldershot: Ashgate, 2009).

34 Some notable examples include María Luisa García Verdugo, *La Lozana Andaluza y la literatura del siglo XVI: la sífilis como enfermedad y metáfora* (Madrid: Editorial Pliegos, 1994); Jesús Ponce Cárdenas, ‘De burlas y enfermedades barrocas: la sífilis en la obra poética de Anastasio Pantaleón de Ribera y Miguel Colodrero de Villalobos’, *Criticón*, 2007, 100, 115–42; Fernández, *La poesía erótica de los Siglos de Oro*.

35 Lía Schwartz and Ignacio Arellano, *Francisco de Quevedo: Un Heráclito cristiano, Canta sola a Lisi y otros poemas* (Barcelona: Crítica, 1998), 447.

36 Ponce Cárdenas, ‘De burlas y enfermedades barrocas’, 115.

37 See his sonnet ‘Musas Italianas y Latinas’, in *Las Obras de Christoval de Castillejo*, 278.

38 The most extensive anthology of mock encomia is *Amphitheatrum Sapientiae Socraticae joco-seriae* (Hannover: 1619).

39 For an excellent overview of examples, see Laurent Pernot, *Epideictic Rhetoric: Questioning the Stakes of Ancient Praise* (Austin, TX: University of Texas Press, 2015).

40 Aulus Gellius, *Attic Nights*, John C. Rolfe (trans) (Cambridge, MA: Harvard UP, 1952), XVII, 12.

41 Arthur Stanley Pease, ‘Things without Honor’, *Classical Philology*, 1926, 21, 29.

42 According to Aristotle, endoxa 'commend themselves to all or to the majority or to the wise’. Aristotle, *Topics* 1, 100b 22-3. Aristotle. *Posterior Analytics. Topica*. Hugh Tredennick and E. S. Forster. (trans.) (Cambridge, MA: Harvard UP, 1960)

43 Pease, ‘Things without Honor’, 30.

44 Rosalie L. Colie, *Paradoxia Epidemica: The Renaissance Tradition of Paradox* (Princeton: Princeton UP, 1966).

45 Some notable classical examples include Polycrates (praise of mice and pebbles), Isocrates (Helen and Busiris), Crates (lentil soup), Dio Chrysostom (parrot, gnat, hair), Favorinus (quartan fever), Synesius (baldness) and Lucian (fly). In the Renaissance, the main representatives would be Erasmus (folly), Daniel Heinsius (ass and louse), Girolamo Cardano (gout).

46 Colie, *Paradoxia Epidemica*, 5.

47
*Ibid.*, 5.

48 ‘*eine Ode an den Guajak*’, in Raphael Finckenstein, *Zur Geschichte der Syphilis* (Wroclaw: Morgenstern, 1870), 38.

49 ‘*un verdadero* loor *vertido a lo burlesco*’, in Rogelio Reyes Cano, *Estudios sobre Cristóbal de Castillejo* (Salamanca: Universidad de Salamanca, 2000), 131.

50 Clara Leonora Nicolay, ‘The Life and Works of Cristóbal de Castillejo’ (dissertation, University of Pennsylvania, 1910), 39.

51 Paracelsus, *Vom Holtz Guaiaco gründlicher heylung* (Nuremberg: F. Peypus, 1529).

52 ‘*donde se unía ya en patética mixtura la forma de lo jocoso con el trasfondo biográfico de lo maldito*’, in Ponce Cárdenas, ‘De burlas y enfermedades barrocas’, 123.

53 Cato, *On Agriculture*, CLVI, in *Cato and Varro on Agriculture*, William Davis Hooper (trans) (Cambridge, MA: Harvard University Press, 1934).

54 Cato’s praise of the cabbage belongs to the large body of texts that are not strictly *laudationes*, but rather medico-botanical treatises. Pythagoras thus praised onions, Moschion radish and Phanias nettle.

55 Juvenal, *Satires*, V. 87, in *Juvenal and Persius*, Susanna Morton Braund (trans) (Cambridge, MA: Harvard University Press, 2004).

56 ‘*una marcada carga cómica por ser alimentos baratos, que provocan gases*’, in María del Rosario Martínez Navarro, ‘Cristóbal de Castillejo y el *milagro americano*: el Palo Santo de Indias y el *mal de bubas* en clave bufonesca, política y anticortesana’, *Temas Americanistas*, 2018, 40, 113. Trans. mine.

57 I am using the categories of Henry W. Wells, *Poetic Imagery Illustrated from Elizabethan Literature* (New York: Russell & Russell, 1961), 189–205.

58 Christopher D. Johnson, *Hyperboles: The Rhetoric of Excess in Baroque Literature and Thought* (Cambridge: Harvard UP, 2010), 16.

59 Goran V. Stanivukovic, ‘“Mounting Above the Truthe”: On Hyperbole in English Renaissance Literature’, *Forum for Modern Language Studies*, 2007, 43, 24.

60 Paula S. DeVos, *Compound Remedies: Galenic Pharmacy from the Ancient Mediterranean to New Spain* (Pittsburgh, PA: University of Pittsburgh Press, 2021), 102.

61 Robert S. Munger, ‘Guaiacum, the Holy Wood from the New World’, *Journal of the History of Medicine and Allied Sciences*, 1949, 4, 207.

62 Ulrich von Hutten, *Of the Wood Called Guaiacum*, Thomas Paynel (trans) (London: Thomas Bertheletus, 1536), Ch. 10.

63 Johnson, ‘N+2, or a Late Renaissance Poetics of Enumeration’, *MLN*, 2012, 127, 1096.

64 On Ovid’s influence on Castillejo, see Steven Wagschal, ‘Ovid Transformed: Cristóbal de Castillejo as Conflicted Cosmopolitan’, in Frederick A. de Armas, ed., *Ovid in the Age of Cervantes* (Toronto: University of Toronto Press, 2010), 175–90.

65 Navarro, ‘Cristóbal de Castillejo y el *milagro americano*’, 110.

66 See Teresa Huguet-Termes, ‘New World Materia Medica in Spanish Renaissance Medicine: From Scholarly Reception to Practical Impact’, *Medical History*, 2001, 45, 359–76. We should also bear in mind John Slater’s warning against the fallacy of the ‘green gold’, that is, the unfeasibility of the theory that the importation of American *materia medica* in the early modern period was a lucrative business. John Slater, ‘The Green Gold Fallacies: Myth and Reality in the Transatlantic Trade in Medicinal Plants (1493–1663)’, in José Pardo-Tomás and Mauricio Sánchez Menchero, eds, *Geografías médicas: Orillas y fronteras culturales de la medicina hispanoamericana (siglos XVI y XVII)* (México: UNAM, 2014), 99–122.

67 An anonymous Augsburg author complained of the high price in his *Ain bevert recept* (1517).

68 Sharon T. Strocchia, *Forgotten Healers: Women and the Pursuit of Health in Late Renaissance Italy* (Cambridge, MA: Harvard University Press, 2019), 203–4.

69 Flood and Shaw, ‘The Price of the Pox in 1527’.

70 Christopher Cook, ‘*An Allegory with Venus and Cupid:* A Story of Syphilis’, *Journal of the Royal Society of Medicine*, 2010, 1, 458.

71
*Sine Cerere et Baccho friget Venus* (‘Without Ceres and Bacchus, Venus freezes’) is a quotation from the Roman comedian Terence, *Eunuchus*, V. 4. It became a proverb in the early modern period.

72 Nicolás Monardes, *Joyfull Newes Out of the Newe Founde Worlde*, John Frampton (trans) (London: William Norton, 1577), footnote 11.

73 Hutten, *Of the Wood Called Guaiacum*, 16.

74
*Ibid.*, 16.

75
*Ibid.*, 20.

76
*Ibid.*, 19.

77
*Ibid.*, 16.

78
*Ibid.*, 61.

79 Hutten, *Of the Wood Called Guaiacum*, 62.

80 William Turner, *The First and Seconde Partes of the Herbal of William Turner* (Cologne: Arnold Birchman, 1568), 34.

81 Alberto Sánchez, ‘Historia y poesía: La historia de Piramo y Tisbe en el *Quijote*’, *Anales Cervantinos*, 1998, 34, 9–22.

82 Thomas Nashe, *Lenten Stuffe* (London: 1599), 24: ‘Physitions deafen our eares with the *Honorificabilitudinitatibus* of their heauenly *Panachæa* their soueraigne *Guiacum*, their glisters, their triacles, their mithridates of fortie severall poysons compacted, their bitter *Rubarbe*, and torturing *Stibium*’.

